# Case report: Complete response in TMB-H advanced uterine clear cell carcinoma: a case analysis of paclitaxel albumin-bound combined with PD-1/CTLA-4 bispecific antibody

**DOI:** 10.3389/fimmu.2024.1486200

**Published:** 2024-12-24

**Authors:** Yue Chen, Wenting Zhou, Yili Wang

**Affiliations:** ^1^ The Oncology Department of the First Affiliated Hospital of Gannan Medical University, Ganzhou, Jiangxi, China; ^2^ Jiangxi Provincial Unit for Clinical Key Oncology Specialty Development, Ganzhou, Jiangxi, China; ^3^ Jiangxi “Flagship” Oncology Department of Synergy for Chinese and Western Medicine, Ganzhou, Jiangxi, China

**Keywords:** PD-1/CTLA-4 bispecific, AK104/cadonilimab, complete response, uterine clear cell carcinoma, immunotherapy

## Abstract

**Background:**

Uterine clear cell carcinoma (UCCC) is a rare and aggressive subtype of endometrial cancer, often presenting at an advanced stage with poor prognosis. Treatment options for advanced or recurrent UCCC are currently limited, especially after platinum-based chemotherapy has failed.

**Case presentation:**

We present the case of a 49-year-old female diagnosed with stage IV uterine clear cell carcinoma. The patient had a history of atrial fibrillation and initially received several surgical interventions and platinum-based chemotherapy, but these treatments resulted in poor outcomes and rapid tumor progression. Genetic testing showed a high tumor mutation burden (TMB-H, 42.24 mutations/Mb) with stable microsatellites and a suspected harmful mutation in the PMS2 gene. After conventional therapies failed, the patient received a combination treatment of cadonilimab (375 mg) and albumin-bound paclitaxel (380 mg) for six cycles. This was followed by cadonilimab monotherapy for maintenance. This treatment regimen led to a complete response (CR), with no detectable abdominal fluid or enlarged lymph nodes by January 4, 2023. The CR status was maintained during a follow-up on April 07, 2024. The adverse effects included severe myelosuppression, mild skin reactions, hypothyroidism, and Grade 3 hyperglycemia, all of which were managed symptomatically.

**Conclusion:**

This case illustrates how effective AK104/Cadonilimab (a PD-1/CTLA-4 bispecific) can be when combined with albumin-bound paclitaxel for treating advanced UCCC, especially in patients who have not responded to standard therapies. The patient’s complete and lasting response shows the potential of PD-1/CTLA-4 bispecific immunotherapy. This suggests that cadonilimab could provide important clinical benefits for patients with advanced or recurrent UCCC.

## Introduction

Uterine clear cell carcinoma (UCCC) is a rare and aggressive subtype of endometrial carcinoma, accounting for about 1 to 5.5% of all endometrial cancers (1, 2). Endometrial clear cell carcinoma (ECCC) is characterized by distinct histopathological features, typically showing clear or eosinophilic cytoplasm along with a high level of nuclear atypia (3, 4). This malignancy is usually diagnosed in postmenopausal women and is associated with a poor prognosis, primarily due to its propensity for deep myometrial invasion, lymphovascular space invasion, and early metastasis (2). The pathogenesis of ECCC is still not fully understood, but recent studies indicate that molecular alterations, particularly TERT promoter mutations and PIK3CA mutations, may play a role (4, 5). Notably, TERT promoter mutations have been identified as independent prognostic factors linked to shorter disease-free and overall survival (6). Despite the rising global incidence of endometrial cancer, there is a significant gap in effective treatment options for advanced or recurrent cases, particularly those resistant to conventional platinum-based chemotherapy (6).

The case report presents a 49-year-old female patient diagnosed with stage IV endometrial clear cell carcinoma. After multiple surgeries and chemotherapy regimens, the patient showed rapid tumor progression. The introduction of cadonilimab with albumin-bound paclitaxel led to complete remission. This outcome highlights the potential effectiveness and safety of PD-1/CTLA-4 bispecific immunotherapy for advanced UCCC. This case emphasizes the need to explore new treatment approaches for this rare and challenging malignancy.

## Case report

**Figure 1:**
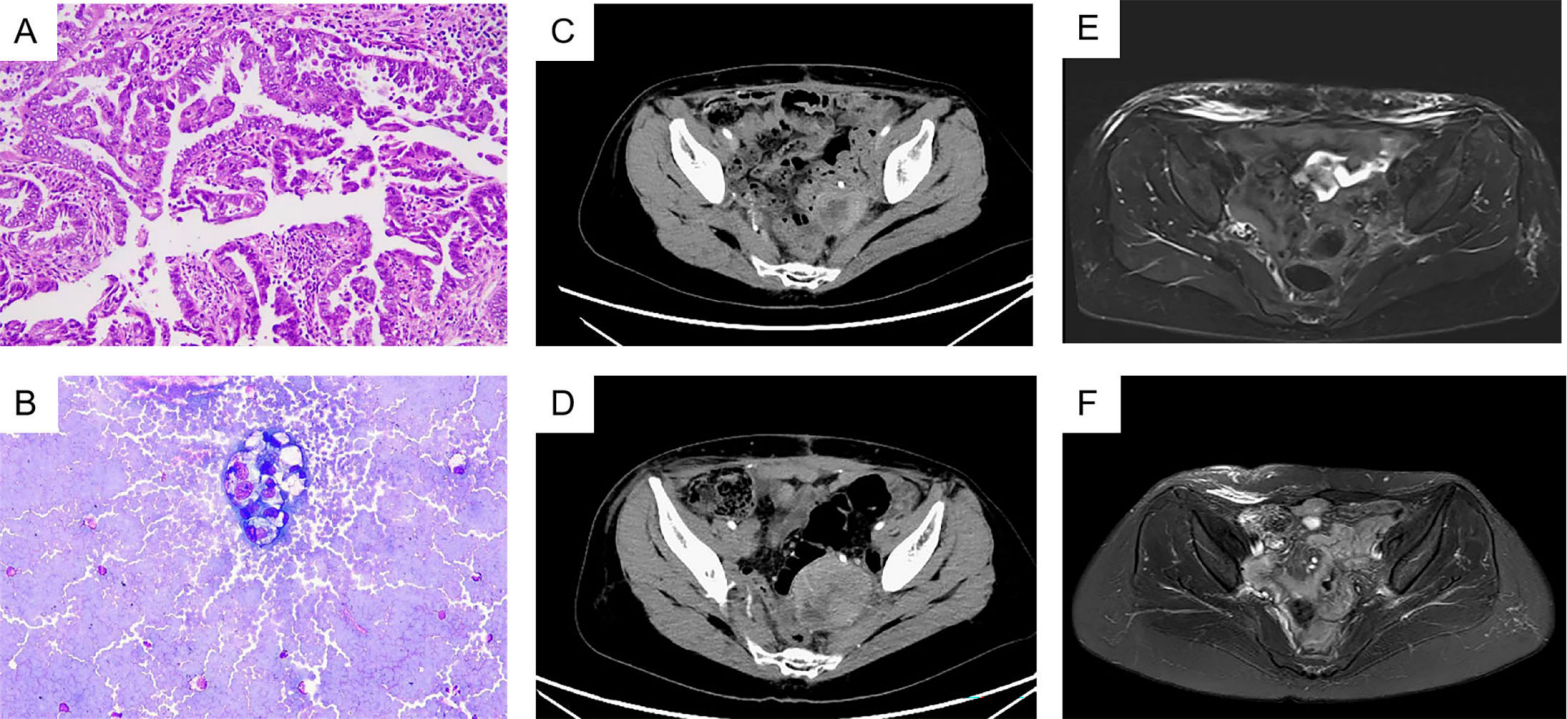
**(A)** HemateinEosin **(B)** Heterotropic cells were seen in ascites. **(C)** 2022-9-27 Total abdominal computerized tomography (CT). **(D)** 2022-10-25 Total abdominal CT, progressive disease. **(E)** 2023-1-4 Total abdominal magnetic resonance, complete remission has been achieved. **(F)** 2024-4-7 Total abdominal MRI, sustained complete response.

A 49-year-old female, measuring 160 cm in height and weighing 45 kg, was admitted to our hospital on October 25, 2022, due to a recurrence of clear cell carcinoma of the uterus more than three years after her initial surgery. The patient first visited our outpatient clinic on December 10, 2019, because of irregular vaginal discharge. Postoperative pathology indicated clear cell carcinoma originating from the lower segment of the uterus (**Figure 1A**), with tumor invasion exceeding half the thickness of the uterine muscle layer, accompanied by abdominal metastasis. The metastatic foci involved the right ovary and the fibrofatty tissue surrounding the appendix, with extensive intravascular cancer emboli observed. Three lymph nodes exhibited evidence of cancer metastasis (**Supplementary Figure 1**). And microscopic examination of the peritoneal flushing solution revealed a small number of cells, including individual heterotypic cell clusters (**Figure 1B**). The high-throughput sequencing test report indicated: 1. High tumor mutation burden (TMB-H, 42.24 Muts/Mb, 99%); 2. Microsatellite stable (MSS); 3. BRCA2 mutation (11.5%); 4. Suspected deleterious mutation of PMS2 germ line (**Supplementary Figures 2A, B**). Diagnosis: 1. Malignant tumor of the uterus (clear cell carcinoma, stage IVB, FIGO 2009); 2. Suspected Lynch syndrome.

From January 6, 2020, to April 6, 2020, the patient underwent six cycles of paclitaxel liposome plus carboplatin adjuvant chemotherapy. During chemotherapy, the patient experienced grade 3 leukopenia and grade 1 transaminase elevation, which improved after symptomatic treatment. Following the identification of BRCA2 mutations, the patient was prescribed oral olaparib for maintenance therapy and attended regular follow-up examinations.

In January 2021, the patient reported abdominal pain. Physical examination showed tenderness in the left lower abdomen and non-depressed edema in both lower limbs. A pelvic MRI and PET-CT scan revealed a solid mass on the left side of the pelvis, indicating a potential tumor recurrence (**Supplementary Figure 3**). After discussions with the multidisciplinary team and taking the patient’s wishes into account, the patient underwent surgery again to remove the lesion. Postoperative pathology revealed lesions in the left sacroiliac ligament consistent with adenocarcinoma metastasis, along with tissue invading the muscle layer at the base of the left bladder. From January 29 to May 21, 2021, the patient received six cycles of adjuvant chemotherapy with paclitaxel liposome and carboplatin. Prophylactic white blood cell treatment was administered during chemotherapy, and the patient experienced no significant adverse reactions. The patient continued taking oral olaparib as ongoing treatment and attended regular follow-up examinations.

**Figure 2:**
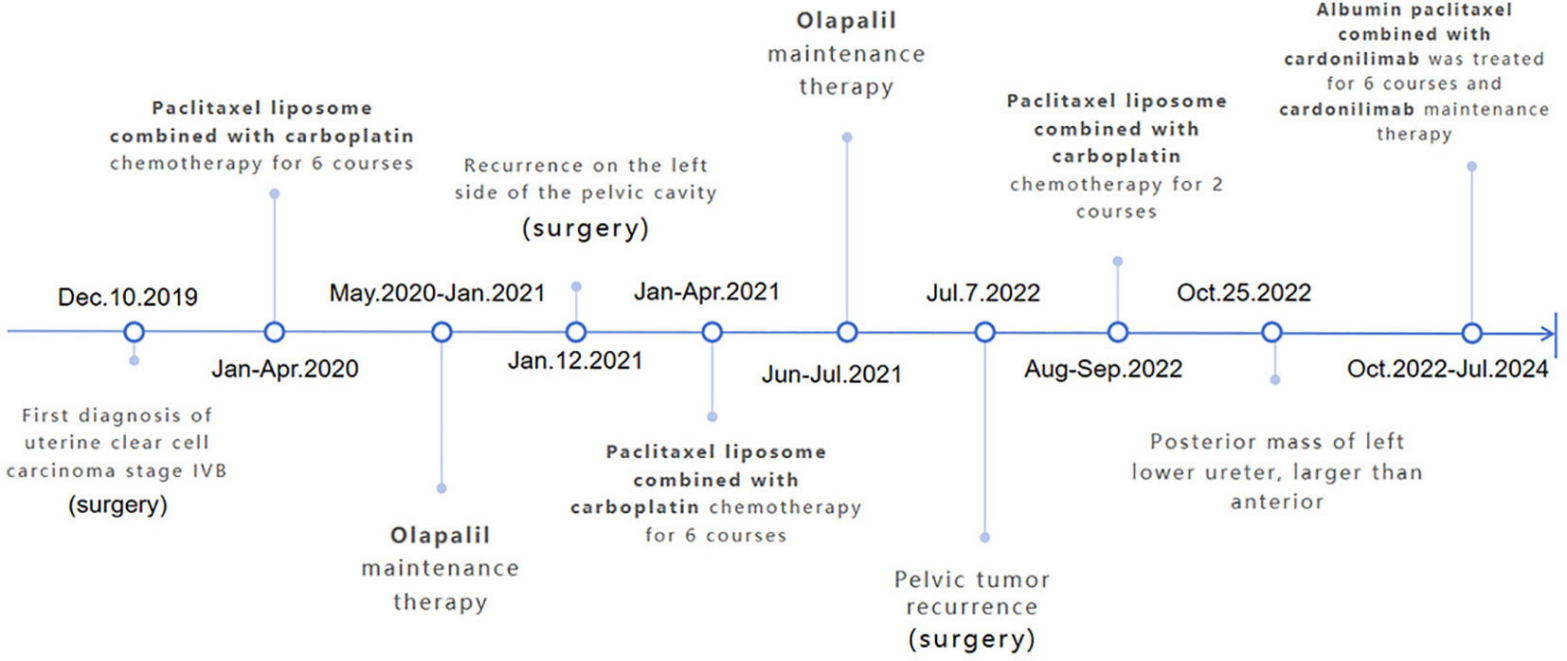
Overall treatment process diagram.

In July 2022, a routine follow-up examination revealed the recurrence of pelvic metastatic tumors in the lower segment of the left ureter (**Supplementary Figure 4**). The patient opted for another surgery, which confirmed clear cell carcinoma in the postoperative pathology of the bladder and lesion. One month after surgery, bilateral inguinal lymphadenopathy was observed. The pathological examination of the inguinal lymph nodes confirmed metastasis. On August 24, 2022, a robot-assisted laparoscopic bilateral inguinal lymph node dissection was performed. This procedure revealed metastasis in 13 of the 45 lymph nodes on the left side, while no metastasis was found in any of the 29 lymph nodes on the right side. The patient received two cycles of paclitaxel liposome plus carboplatin chemotherapy after surgery. After surgery, the patient experienced persistent gross hematuria in the ureter, and a CT scan revealed a posterior mass in the lower segment of the left ureter, which had increased in size (54mm x 65mm) compared to before (**Figures 1C, D**). After obtaining informed consent from the patient and their family, the patient began combination chemotherapy with paclitaxel albumin-bound (380 mg, 240mg/m2) and cadonilimab (375 mg, 6mg/kg) immunotherapy on October 28, 2022, administered every three weeks for six cycles. On February 21, 2023, the patient continued with cadonilimab. The patient continued to use cadonilimab monotherapy for a total of 20 months until July 8, 2024, when the drug was discontinued due to adverse reactions (**Figure 2**).

Following three cycles of treatment with cadonilimab and albumin-bound paclitaxel, the patient showed no signs of ascites or enlarged lymph nodes behind the peritoneum. On January 4, 2023, the treatment response was evaluated as complete remission (CR) (**Figure 1E**). The patient continued regular follow-up examinations and maintained complete remission until the most recent follow-up on April 7, 2024 (**Figure 1F**). During treatment, the adverse reactions associated with immunotherapy included neutropenia following the first three cycles of combined immunotherapy and chemotherapy, which improved with long-acting white blood cell boosters and subsequent preventive measures; mild rash and itching after three cycles, which resolved with skincare and topical ointment; hypothyroidism on June 28, 2023, treated with levothyroxine sodium tablets 50 µg/day, with normal thyroid function on July 15; and hyperglycemia on July 16, 2024, with random blood glucose of 26 mmol/L, which improved with insulin therapy. The patient discontinued maintenance medication due to experiencing grade 3 hyperglycemia during the last treatment cycle.

## Discussion

This case involves a 49-year-old woman with recurrent uterine clear cell carcinoma (UCCC) and discusses key points in managing this rare and aggressive type of endometrial cancer. This case is significant because the patient has a long history of surgeries and chemotherapy, ultimately leading to a positive response to a new immunotherapy treatment.

UCCC, a type II endometrial cancer, is associated with various factors, including the use of diethylstilbestrol during pregnancy, genetic susceptibility, microsatellite repeat instability, Bcl-2 overexpression, p53 gene mutation, human papillomavirus infection, and environmental factors (7). UCCC is frequently linked to a poor prognosis due to its aggressive nature and early metastasis, with factors such as patient age, tumor size, stage, surgical intervention, number of detected lymph nodes, lymph node metastasis, radiotherapy, and chemotherapy all correlating with its prognosis (8, 9). Current literature shows that UCCC shares molecular characteristics with other high-grade endometrial cancers, including serous carcinoma, characterized by frequent mutations in the p53 gene and alterations in the PI3K/AKT pathway (10, 11). Another meta-analysis showed that 58% of UCCCs had genetic deletions of PETN, while P53 mutations accounted for 23%, and 38% were positive for P16, and only 8% were positive for PDL1. In dMMR, one protein was missing in 40% of cases, and two proteins were missing in 21% of cases (12). Since the molecular mechanism of UCCC is not well understood, research is actively focused on determining the most effective treatment strategies for advanced or recurrent cases.

The patient’s initial treatment included extensive surgeries such as a total hysterectomy, bilateral salpingo-oophorectomy, and several lymphadenectomies, followed by multiple cycles of chemotherapy with paclitaxel and carboplatin. Despite these aggressive treatments, the patient had several recurrences, which highlights the challenges of managing advanced UCCC. Similarly, other studies indicate that traditional chemotherapy often yields limited success in preventing recurrence and improving overall survival in UCCC patients. This is supported by other studies that show traditional chemotherapy often fails to effectively prevent recurrence and enhance overall survival in UCCC patients (13).

This patient was diagnosed with stage IV B endometrial clear cell carcinoma, which progressed after the initial surgery on December 12, 2019, and multiple subsequent treatments. The patient’s genetic test results indicated a high tumor mutation burden (TMB-H, 42.24 Muts/Mb, 99%), placing her in a group that could potentially benefit from immunotherapy (14). PMS2 was suspected to have deleterious mutations in the germline within the tumor tissue, suggesting that the suspected Lynch syndrome might indicate the effectiveness and durability of immunotherapy (15). The incidence of Lynch syndrome with PMS2 deletion is relatively low and may be linked to mechanisms such as MLH1 methylation (16). However, further investigation was not pursued due to the patient’s personal reasons, despite the presence of other malignancies in her family. Dual immunotherapy, combining anti-programmed cell death-1/ligand 1 (anti-PD-1/L1) with either anti-cytotoxic T-lymphocyte-associated protein 4 (anti-CTLA-4), offers advantages over immune checkpoint inhibitor (ICI) monotherapy in terms of overall survival (OS), objective response rate (ORR), and progression-free survival (PFS) (17). AK104/Cadonilimab, as a PD-1/CTLA-4 bispecific antibody, has a unique double antibody structure that simultaneously blocks two different inhibition sites (18), making it more convenient to use. This case further verified the effectiveness of dual antibody therapy in the treatment of UCCC.

The use of immune checkpoint inhibitors like Cadonilimab, together with chemotherapy, is a promising advancement in treating recurrent UCCC. In this case, the patient achieved complete remission (CR) after six cycles of Cadonilimab with albumin-bound paclitaxel, followed by maintenance therapy with Cadonilimab alone. Although the patient chose 6mg/kg every 3 weeks due to economic and concerns about the side effects of the drug, which was different from the recommended 6mg/kg every 2 weeks or 10mg/kg every 3 weeks (19), satisfactory results were still obtained. This outcome is especially important because conventional therapies have shown limited effectiveness in similar cases. Recent studies have shown that immune checkpoint inhibitors can enhance the anti-tumor immune response, demonstrating their potential in treating various gynecologic malignancies (18).

Although the patient experienced severe leukopenia and hyperglycemia during treatment, both conditions improved with symptomatic medication. However, the patient

ultimately decided to discontinue the medication due to concerns about hyperglycemia. The toxic and side effects associated with immunotherapy in this patients treated with cadonilimab are basically consistent with the adverse effects reported in clinical studies related to cadonilimab (19). The patient was satisfied with the effect of complete remission, but unfortunately, subsequent adverse reactions caused the treatment to be unable to continue. The patient’s response to Cadonilimab and paclitaxel highlights the importance of personalized treatment strategies for managing recurrent UCCC. The use of immune checkpoint inhibitors, either alone or in combination with chemotherapy, may provide a viable option for patients who do not respond to conventional cancer treatments. Nevertheless, further research is necessary to establish standardized protocols and identify biomarkers that can predict responses to immunotherapy in patients with UCCC (20). To better understand adverse reactions, larger sample sizes are needed for long-term observation.

## Conclusion

In conclusion, this case highlights the challenges of managing recurrent UCC and suggests that incorporating immune checkpoint inhibitors into the treatment regimen could be beneficial. This patient’s successful response indicates that combining Cadonilimab with chemotherapy may open new therapeutic options for those with advanced or recurrent UCCC. Ongoing research and clinical trials are vital to confirm these findings and enhance treatment strategies for this aggressive form of UCCC.

## Data Availability

The original contributions presented in the study are included in the article/**Supplementary Material**. Further inquiries can be directed to the corresponding author.
