# Supplementary figures and images for: Case report: Complete response in TMB-H advanced uterine clear cell carcinoma: a case analysis of paclitaxel albumin-bound combined with PD-1/CTLA-4 bispecific antibody
Source: Front Immunol. 2024 Dec 24;15:1486200. doi: 10.3389/fimmu.2024.1486200 (PMC11703853; doi:10.3389/fimmu.2024.1486200)

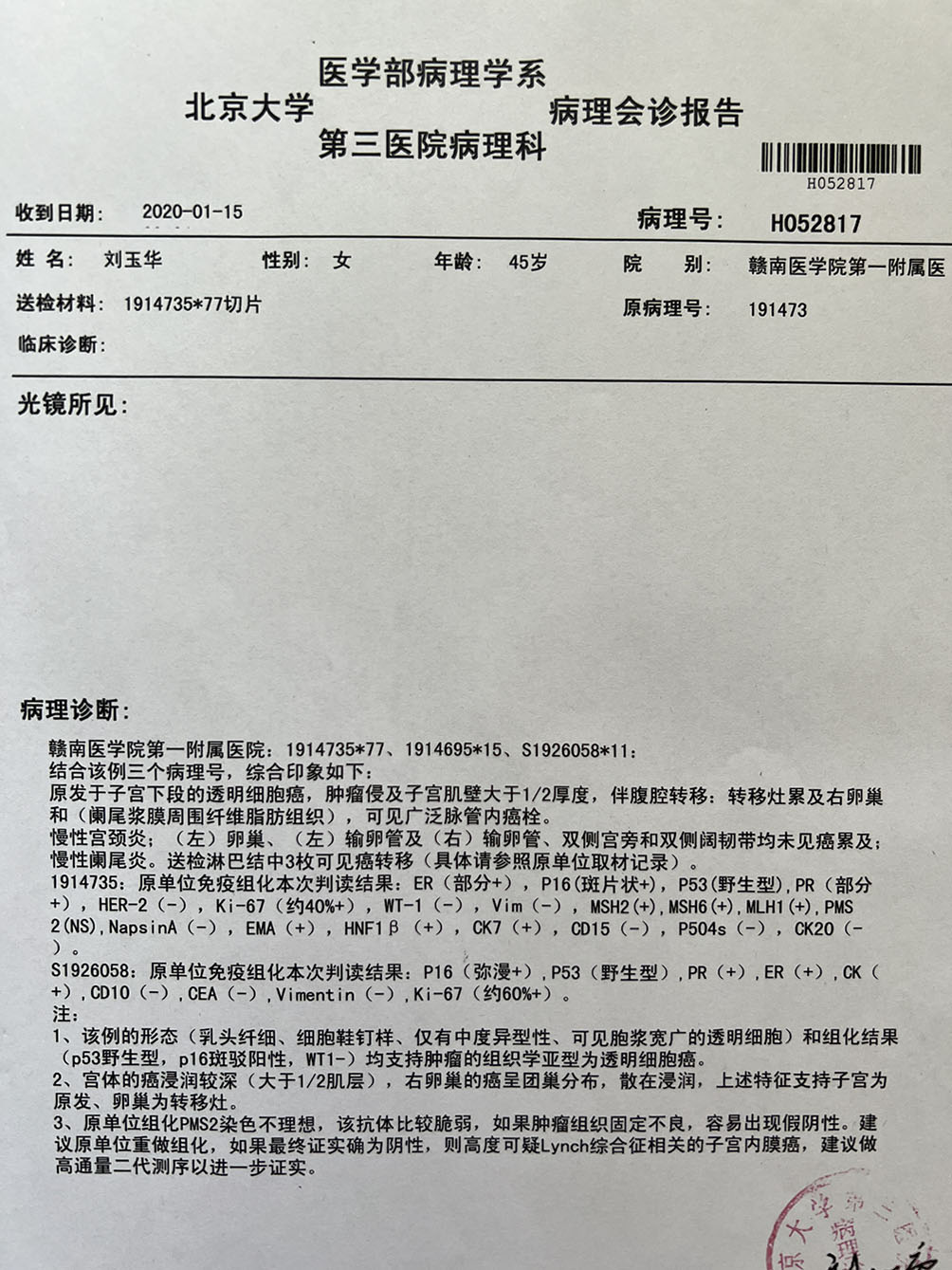

Supplement: Supplementary file 1 [file Presentation1.zip › Editorial 1.JPEG]

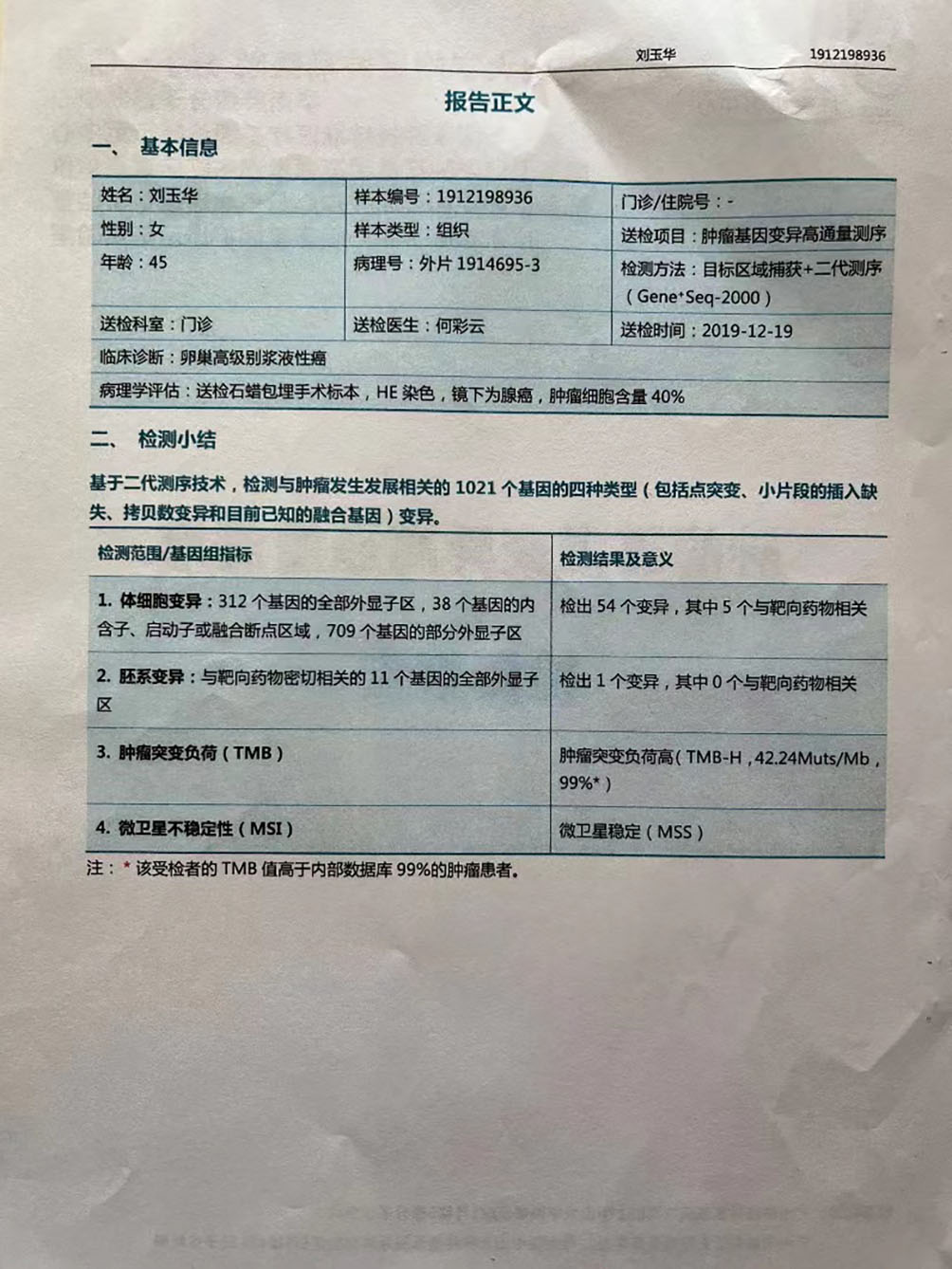

Supplement: Supplementary file 1 [file Presentation1.zip › Editorial 2.JPEG]

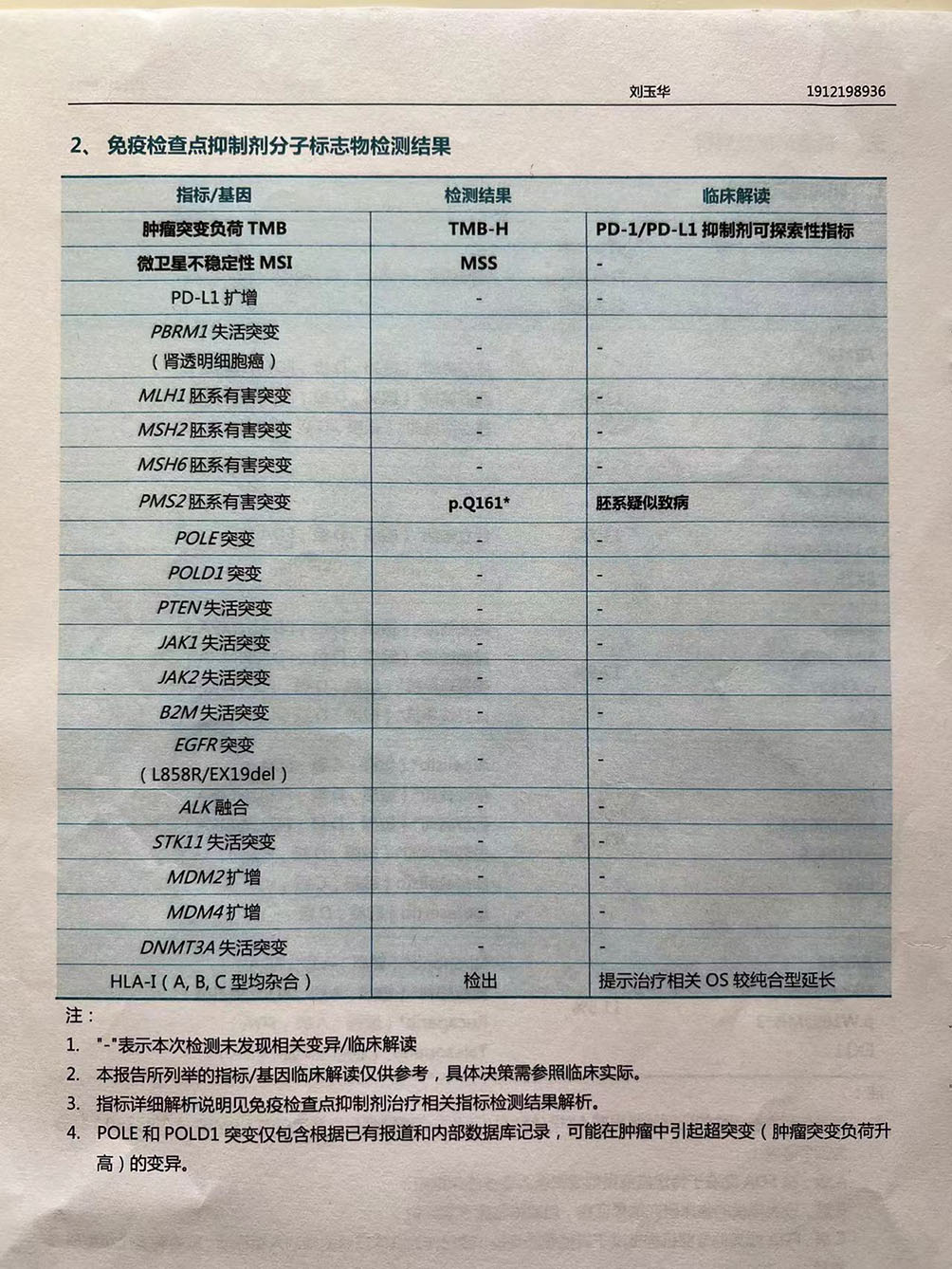

Supplement: Supplementary file 1 [file Presentation1.zip › Editorial 3.JPEG]

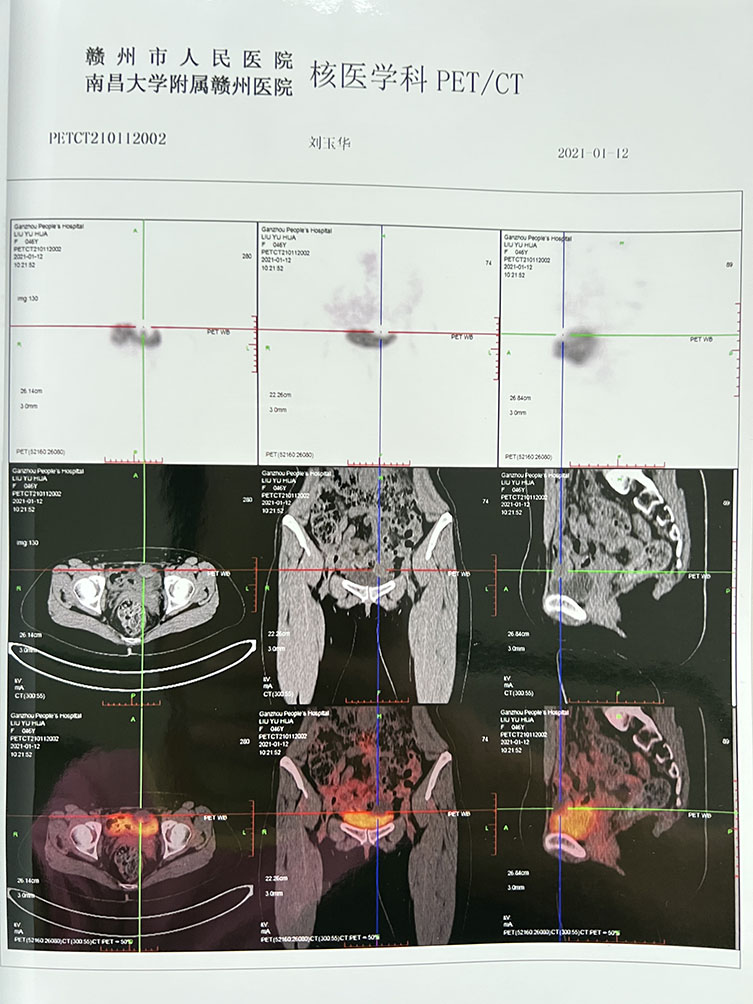

Supplement: Supplementary file 1 [file Presentation1.zip › Editorial 4.JPEG]

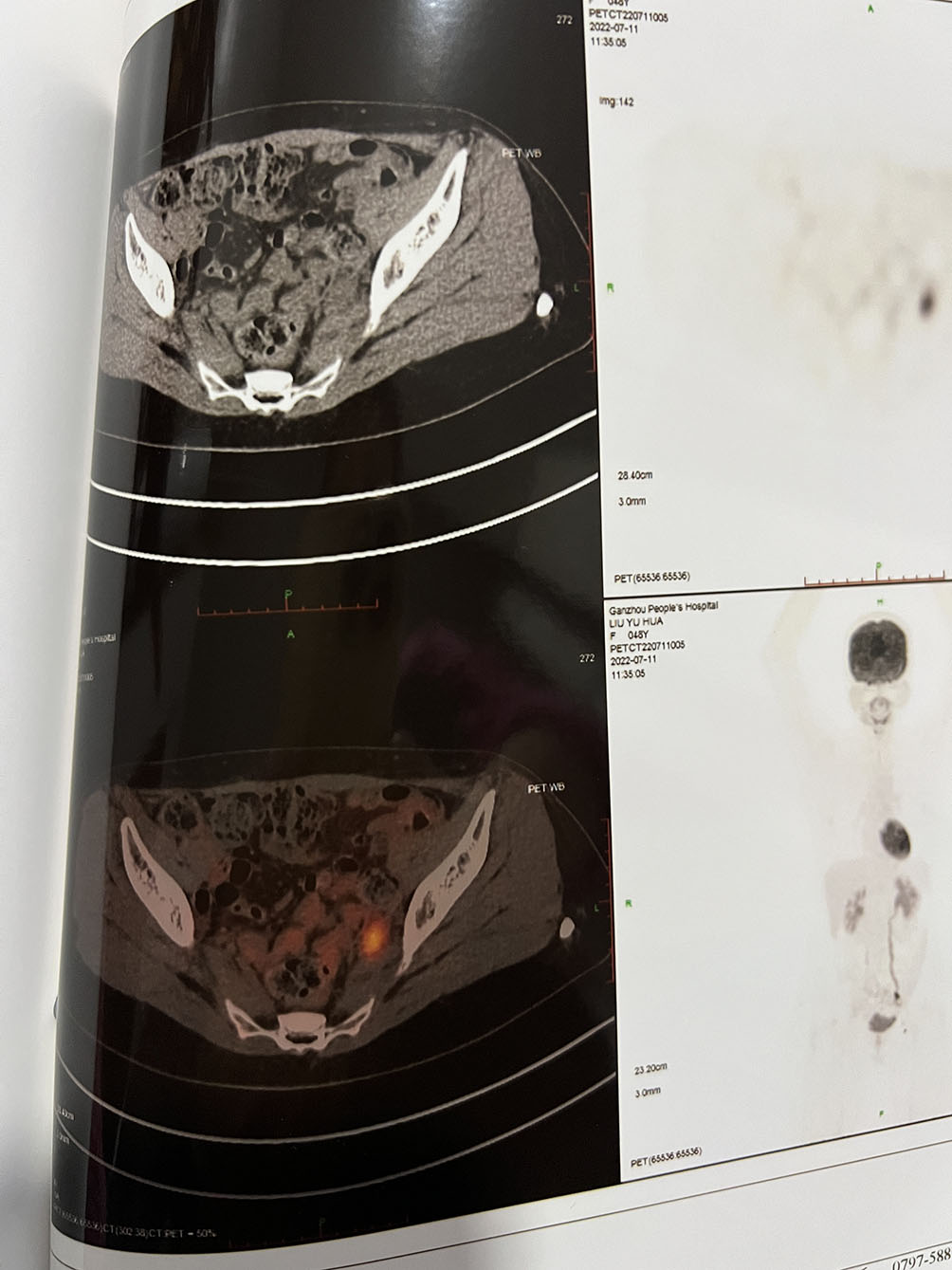

Supplement: Supplementary file 1 [file Presentation1.zip › Editorial 5.JPEG]

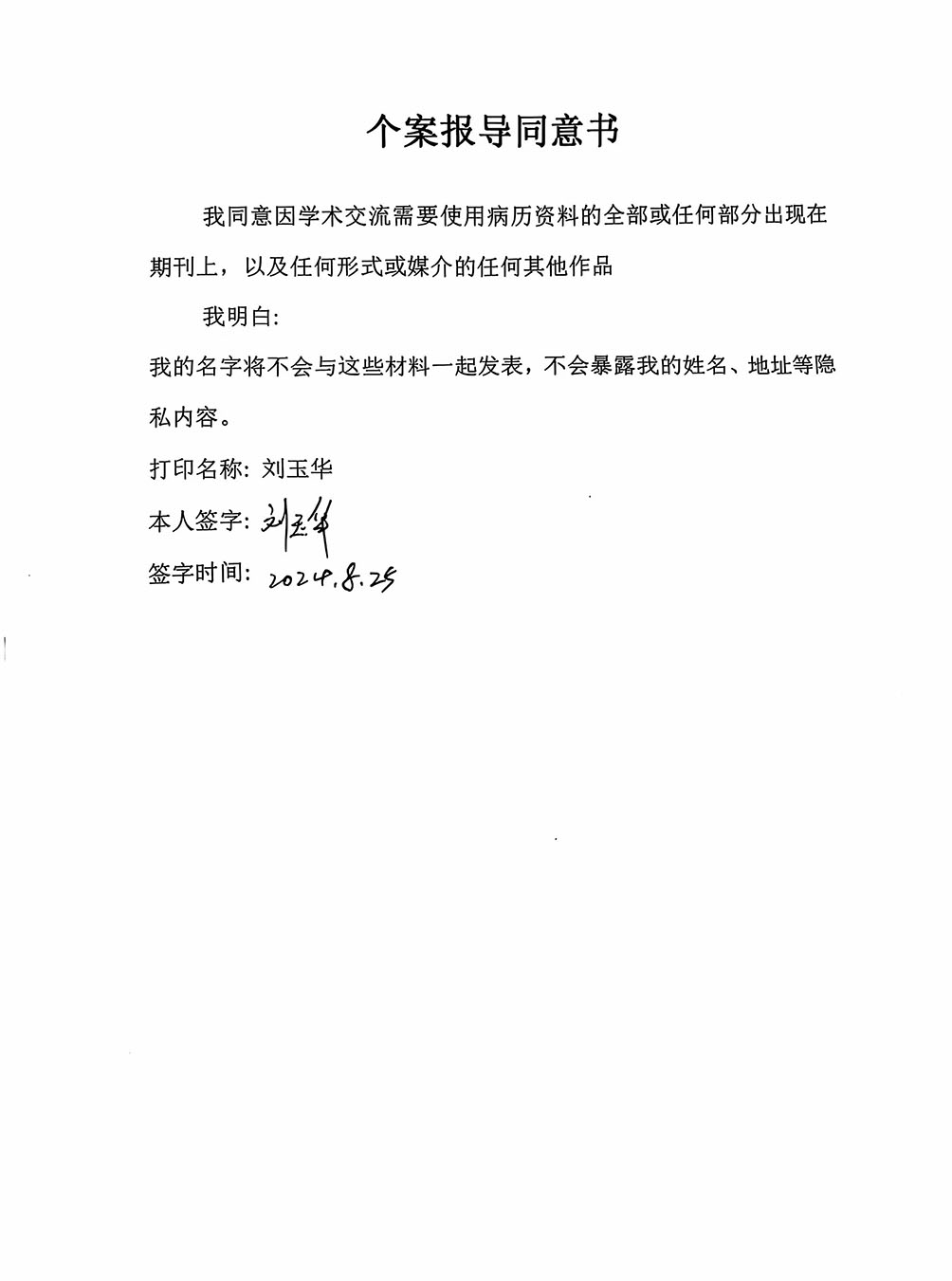

Supplement: Supplementary file 1 [file Presentation1.zip › Editorial 6.JPEG]
